# Factors Associated with EEG Slowing in Individuals with Parkinson's Disease

**DOI:** 10.4314/ejhs.v32i1.9

**Published:** 2022-01

**Authors:** Biniyam A Ayele, Heera Tesfaye, Mehila Z Wuhib, Guta Zenebe

**Affiliations:** 1 MD, Assistant Professor of Neurology Department of Neurology, Addis Ababa University, Addis Ababa, Ethiop; 2 MD, Yehuleshet Specialty Clinic, Addis Ababa, Ethiopia; 3 Mehila Z. Wuhib, MD, Yehuleshet Specialty Clinic; 4 Honorary Assistant Professor of Neurology Department of Neurology, College of Health Sciences Addis Ababa University, Addis Ababa, Ethiopia; 5 Guta Z. Metaferia, MD, Associate Professor of Neurology, Department of Neurology, School of Medicine College of Health Sciences, Addis Ababa University Addis Ababa Ethiopia

**Keywords:** Electroencephalograph, Parkinson's disease, hypovitaminosis D, forgetfulness, Ethiopia

## Abstract

**Background:**

A plethora of scientific studies has shown diffuse slowing on electroencephalograph (EEG) study is a frequent occurrence in Parkinson's disease (PD) patients, compared to the healthy controls. Little is known about EEG slowing and PD in the sub-Saharan Africa, especially in Ethiopia. The objective of this study was to assess factors associated with EEG slowing in individuals with Parkinson's disease.

**Method:**

A cross-sectional observational study was conducted in 40 PD patients at Yehuleshet Specialty Clinic, Addis Ababa, Ethiopia. Both descriptive and analytical statistics were used to analyze the data.

**Results:**

Total of 40 patients with PD was included in the present survey. The median age was 66 (IQR: 52.5 – 72.5 years) and young onset PD accounted 20%. Males accounted for twothird of the participants. Diffuse EEG slowing was observed in 52.5% (n=21) of participants. Majority (85%) were on levodopa treatment. Hypovitaminosis D was observed in 93.1% of the study participants. White matter hyperintensity (WMH) and global brain atrophy were seen in 47.5% and 27.5% respectively. Even though statistically not significant, PD patients with EEG slowing, reported more forgetfulness and had WMH on their brain MRI, compared to those with normal EEG. Age was associated with diffuse EEG slowing when adjusted for forgetfulness and WMH (Adjusted OR 1.18 95% CI (1.01 – 1.37) p=0.03).

**Conclusion:**

The present study indicates high prevalence of diffuse EEG slowing in PD patients. Age was associated with diffuse EEG slowing. Higher proportion of patients with EEG slowing reported forgetfulness and hypovitaminosis D compared to those with normal EEG recordings.

## Introduction

Parkinson's disease (PD) is the commonest neurodegenerative disorder, second to Alzheimer's disease (AD) (1). The global burden of PD has more than doubled as a result of increasing numbers of older people, with potential contributions from longer disease duration, and environmental factors (1). Electroencephalography (EEG) is a type of neurophysiological assessment using arrays of electrodes placed across the scalp to record cortical activities in real time; EEG has a better spatial resolution ability in detecting neuronal cellular function compared to structural neuroimaging (2). Thus, it has a unique contribution in assessing the occurrence and progression of the non-motor symptoms of Parkinson's disease such as cognitive impairment (3–5).

The correlations between diffuse EEG slowing and cognitive impairment in PD patients were widely studied (3–7). Furthermore, Barcelon et al. 2019 (5), investigated the role of semi-quantitative EEG analysis to help us to differentiate Parkinson's disease from atypical parkinsonian disorders such as, multiple system atrophy (MSA), progressive supranuclear palsy (PSP), and Corticobasal degeneration (CBD). Accordingly, the authors reported the modified grand total EEG (GTE) score can distinguish patients with PD from those with CBD, PSP or MSA at a cut-off score of 9 with excellent sensitivity but poor specificity (5). Plethora of scientific studies have showed higher prevalence of diffuse slowing EEG pattern in PD patients compared to healthy controls (2,3,8–11). Diffuse EEG slowing has been associated with the presence of global cortical atrophy, dementia, and in patients with advanced age (2,9,12,13). The association between dopaminergic neuronal degeneration in the substantia nigra and EEG slowing is still unknown. EEG is only helpful in recording the functions of the cortical neurons, not the subcortical neurons such as dopaminergic neurons in the substantia nigra (14).

To our best knowledge, this is the first report from the sub-Saharan African (SSA) on diffuse EEG slowing in African patients with PD. The paucity of the scientific reports on this topic from SSA is likely related to the universal absence of advanced electrodiagnostic tests such as EEG in majority of African countries and lack of trained health care professionals. Thus, the objective of the present study was to determine factors associated with diffuse EEG slowing in Ethiopian patients with Parkinson's disease.

## Materials and Methods

**Study objective and study setting**: The study was conducted at the outpatient neurology clinics of Yehuleshet Specialty Clinic (YSC) in Addis Ababa, Ethiopia. Yehuleshet Specialty Clinic is a specialty clinic located at the heart of Addis Ababa. The clinic is equipped with 4 latest 32-channel Nicolet video EEG machines 2019 model, seven EEG-trained nurses, and 0.35 tesla magnetic resonance image (MRI) machine.

**Study period and design**: A cross-sectional observational study was conducted between May 2020 and February 2021. A total of 40 patients with confirmed PD and had at least one EEG record were included in the study analysis. All patients were evaluated and diagnosed using UK Parkinson's Disease Society Brain Bank (UKBB).

**Data collection tool and procedure**: A structured questionnaire was used in assessing the demography and clinical characteristics of PD patients. All the patients were clinically evaluated and questionnaires were administered to each participant by certified neurologists. Additional data including investigations and images were extracted from individual patient's medical recorder data.

**Electroencephalogram recording**: EEG recording was done using NicoletOne video EEG 2019 Netus machine based on 10–20 international system. The procedure of EEG recording follows an international protocol (15,16). The EEG recording was performed by trained EEG technicians at electrodiagnostic unit in Yehuleshet Specialty Clinic. The EEG technician instructed the patient to sit upright in a quiet and dimly shielded room with eyes closed to attain a state of relaxed wakefulness. The patients were instructed not to fidget, talk, or move to avoid movement artifacts during the recording process. During the recording process, hyperventilation and photic stimulation were used as an activation procedure. Each EEG recording lasted 30 minutes.

**EEG interpretation and reporting**: All the recorded EEG tracings were interpreted and reported by two independent board-certified neurologists, who are not part of the present study, but currently working at YSC. The experts interpreted and reported the EEG findings based on an internationally accepted protocol, which was implemented in the clinic (15,16). The experts report included the following information for every EEG tracing: demographic data; description of the EEG background rhythm (delta, theta, alpha, and beta); and presence/or absence of epileptiform discharges/ or dysrhythmia.

**Data analysis**: Variables were described using means, median, frequency, percentile, and standard deviation, and interquartile range. Associations were done using chi square or Fisher exact test, logistic regression analysis and results were presented using odds ratio (OR), and p value was set at < 0.05 as statistically significant.

## Results

**Baseline characteristics and EEG findings of study participants**: In the present study, total of 40 PD patients were included in the analysis. The median age was 66 (IQR: 52.5 – 72.5 years). Young onset PD (≤ 50 years) accounted 20%. Males accounted for two-third of the study participants. The median duration of illness was 2.0 (IQR: 1 – 3 years) and Hoehn and Yahr (H & Y) stage 1 & 2 accounted 70%. Of the forty EEG recordings included in the present survey, 52.5% (n=21/40) showed generalized background slowing (delta and theta waves). The median serum vitamin D level was 10.6 (8.1 – 18.3) ng/mL. The prevalence of vitamin D insufficiency (level < 30 ng/mL) was 93.1%. Thirty-four (85%) of the patients were on levodopa monotherapy, and 15% were on combination of levodopa and anticholinergic.

Subjective forgetfulness, hallucination, and constipation were reported in 62.5%, 30%, and 70% of study participants respectively. Hypertension was the commonest comorbid medical illness (25%). Neuroimaging studies showed white matter hyperintensity and global brain atrophy in 47.5% and 27.5% of the study participants respectively. Out of forty PD patients, five (12.5%) showed positive serum venereal disease research laboratory (VDRL) test, indicating a probable syphilis infection and 2.5% (n=1) had HIV infection. Anemia was observed in 15% of study participants (Table 1).

**Table 1:** Baseline characteristics and EEG findings of the study participants (n=40)

Characteristics	Values
Age in years (median, IQR)	66 (52.5 – 72.5)
Young onset PD (n, %)	8 (20)
Male (n, %)	27 (67.5)
Duration of illness in years (median, IQR)	2.0 (1 – 3)
HY stage 1 & 2 (n, %)	28 (70)
Vitamin D level (median, IQR)	10.6 (8.1 – 18.3)ng/mL
Hemoglobin level (mean, 1SD)	14.3 (1.8)g/mL
Levodopa treatment (n, %)	34 (85)
Anticholinergic treatment (n, %)	6 (15)
Forgetfulness (n, %)	25 (62.5)
Hallucination (n, %)	12 (30)
Constipation (n, %)	28 (70)
Hypertension (n, %)	10 (25)
Diabetes mellitus (n, %)	4 (10)
Dyslipidemia (n, %)	3 (7.5)
Brain MRI findings (n, %)	
Normal	13 (32.5)
Non-specific white matter hyperintensity	19 (47.5)
Global brain atrophy	11 (27.5)
Incidental findings	2 (5)
Electroencephalograph findings (n, %)	
Normal background	19 (47.5)
Generalized background slowing	21 (52.5)
HIV infection (n, %)	1 (2.5)
Syphilis infection (n, %)	5 (12.5)

¶SD: Standard deviation; IQR: Interquartile range; N: Frequency; H & Y: Hoehn and Yahr

**Factors associated with EEG slowing in PD patients**: In the present survey, majority of the young onset PD patients had normal EEG background rhythm. No significant difference was observed between the EEG slowing and gender (p=0.12) and disease stage (p=0.26). Similar result was observed between EEG slowing and use of anticholinergic medications (p=0.90), forgetfulness (p=0.57), and comorbid hypertension (p=0.72) (Table 2). Even though statistically not significant, the presences of white matter hyperintensity on brain MRI (p=0.19), vitamin D insufficiency (p=0.22), and the presence of anemia (p=0.18) were shown positive trends with diffuse EEG slowing (Table 2). In the present survey, study participants with EEG slowing have lower mean serum vitamin D level compared to those with normal EEG (14.7 ng/mL vs. 13.6 ng/mL, p=0.74). Though not statistically significant, the serum vitamin D level declines with increasing disease stages (p=0.08). Furthermore, lower mean vitamin D was observed among individuals with white matter hyperintensity (WMH) compared to those with non-WMH (13.8 ng/mL vs. 14.8 ng/mL, p=0.09 respectively). Similarly, the mean hemoglobin level was lower in individuals with WMH compared to those with no-WMH (13.6 g/mL vs. 14.7 g/mL, p=0.08 respectively). White matter hyperintensity was observed more in PD patients with EEG slowing compared to those with normal EEG (30% vs. 17.5%, p=0.19).

**Table 2:** Factors associated with EEG slowing in PD patients

Variable	EEG slowing N (%)	No-EEG slowing N (%)	Fisher Exact Test
**Age Below 50 years**	2 (5)	6 (15)	0.12
**Age Above 50 years**	19 (47.5)	13 (32.5)	
**Male**	14 (35)	13 (32.5)	0.91
**Female**	7 (17.5)	6 (15)	
**H & Y stage**			
**stage 1**	4 (10)	5 (12.5)	0.26
**stage 2**	11 (27.5)	8 (20)	
**stage 3**	3 (7.5)	6 (15)	
**stage 4**	3 (7.5)	0 (0)	
**Anticholinergic therapy**			
**Yes**	3 (7.5)	3 (7.5)	0.90
**No**	18 (45)	16 (40)	
**Forgetfulness**	14 (35)	11 (27.5)	0.57
**No-forgetfulness**	7 (17.5)	8 (20)	
**Hypertension**			
**Yes**	6 (15)	4 (10)	0.72
**No**	15 (37.5)	15 (37.5)	
**Brain MRI**			
**WMH**	12 (30)	7 (17.5)	0.19
**Normal/ or non-WMH**	9 (22.5)	12 (30)	
**Syphilis infection**			
**Yes**	3 (7.5)	2 (5)	0.90
**No**	18 (45)	17 (42.5)	
**Vitamin D level**			
**Insufficiency**	15 (51.7)	12 (41.4)	0.22
**Normal** **Normal**	0 (0)	2 (6.9)	
**Anemia**	5 (12.5)	1 (2.5)	0.18

¶MRI: Magnetic resonance image; WMH: White mater hyperintensity; N: Frequency; H & Y: Hoehn and Yahr

**Logistic regression analysis of EEG slowing and covariates**: Both in univariate and multivariate logistic regression analysis, age of the patients was associated with diffuse background EEG slowing when adjusted for subjective complains of forgetfulness and white matter hyperintensity on brain MRI (Adjusted OR 1.18 95% CI (1.01 – 1.37) p=0.03). No correlation was observed between generalized slowing on EEG and subjective complaint of forgetfulness and white matter hyperintensity on brain imaging (Table 3).

**Table 3:** Logistics regression analysis of EEG slowing and covariates in study participants

Covariates	Crude OR	95% CI	P value	Adjusted OR	95% CI	P value
Age	1.07	1.01 – 1.14	0.02	1.18	1.01 – 1.37	0.03
Forgetfulness						
No	Ref.					
Yes	1.46	0.40 – 5.26	0.57	3.53	0.31 – 40.11	0.31
WMH						
No	Ref.					
Yes	2.29	0.64 – 8.15	0.20	3.49	0.50 – 24.39	0.21

¶COR: Crude odds ratio; AOR: Adjusted odds ratio; CI: Confidence interval; WMH: White matter hyperintensity; Ref.: Reference

## Discussion

To our knowledge this is the first study to report on factors associated with EEG slowing in Ethiopian PD patients. Accordingly, in the present survey, males accounted for the majority of the study participants; which is consistent with previous local, regional, and global figures (1,17–19). The mean age was in sixth decades, which is comparable to the previous report from Ethiopia (20–22). More than half of the patients had diffuse EEG slowing. Age of the patients was associated with of EEG slowing. This finding is consistent with previous studies (3,6,11,12,23,24). In addition, even though statistically not significant, those patients with hypovitaminosis D, PD patients who reported subjective forgetfulness and those with white matter hyperintensity on brain MRI tended to have EEG slowing compared to those without the above listed disorders.

Electroencephalography is a non-invasive, simple to use, and cost effective technique that records the electrical activity produced by the cortical neurons in the brain; and it has a good temporal resolution and high test-retest reliability, which is increasingly recognized as a fundamental hallmark of cortical integrative functions (8). The present study showed high prevalence of EEG slowing in Ethiopian PD patients. This finding is in congruent with similar report from Finland, which showed high prevalence of EEG slowing among PD patients compared to healthy controls (3). Furthermore, the study reported severe slowing (delta background) was observed among demented PD patients. Likewise, previous studies have consistently showed the direct correlation between the presences of diffuse EEG slowing and cognitive decline in patients with PD (10,12,13,25). In this survey, even though statistically not significant, PD patients who reported subjective forgetfulness tended to have EEG slowing compared to those with noforgetfulness. Therefore, it's commendable to screen PD patients with EEG slowing for cognitive impairment. Thus, such physiological biomarkers are vital to detect and manage PDrelated dementia timely.

In this survey, age was significantly associated with EEG slowing. This is in congruent with previous reports that showed older PD patients tends to have more EEG slowing compared to young PD patients (3,6,8,13,23). Furthermore, diffuse EEG slowing is an indicator of diffuse cortical neuronal cells dysfunction. Thus, patients with diffuse EEG slowing could clinically present with features related to dementia such as forgetfulness, visuospatial disturbance, and wide range of executive function impairment (3,8,12,13,23). Therefore, it is important to screen older PD patients with electroencephalograph, as this will help the treating physician to identify those at risk of PD related cognitive decline.

In the present survey, no association was found between hypovitaminosis D and EEG slowing. However, the prevalence of vitamin D deficiency was higher among PD patients with EEG slowing compared to those with normal EEG tracing. In addition, we have observed gradual decline in serum vitamin D level as disease stages progresses. These finding could be explained by the fact that, in advanced disease stages most of patients will become immobile and confined to their home, which will reduce significantly their exposure to sun light and predispose them to have hypovitaminosis D. However, the lack of association between hypovitaminosis D and EEG slowing in this study could be due to small sample size and lack of healthy control group. Nevertheless, this results highlights on the need to further dig in to the possible association between level of serum vitamin D and EEG pattern in PD patients. As hypovitaminosis D is one of the few potentially reversible metabolic disorders (26). Currently scientific studies have demonstrated low serum vitamin D level may predict an elevated risk of Parkinson's disease incidence (26,27). Furthermore, individuals with PD have lower levels of serum vitamin D than their healthy controls; in addition, hypovitaminosis D has been associated with endothelial dysfunction which may play an important role in the pathogenesis and progression of PD (28).

The limitations of this study include the lack of healthy age and sex matched control group to compare the findings associated with serum vitamin D level. This is one of the most important limitations to the present survey, because previous studies from Ethiopia on vitamin D have shown relatively lower level of serum vitamin D among both healthy and ill Ethiopians, despite abundance of sunlight year round (29–30). However, patients with PD could be uniquely prone to low vitamin D as the disease stage progress. This is mainly because of immobility and reduced exposure to adequate sun shine. The second limitation was small sample size, which could result in lack of adequate power to get statistically significant association between dependent and independent variables.

In summary, the present study indicates high prevalence of EEG slowing among PD patients. Age was associated with diffuse EEG slowing. Even though not significant, higher proportion of patients with EEG slowing reported forgetfulness and hypovitaminosis D compared to those with normal EEG tracing. Therefore, we recommend conducting future control study to consolidate the current findings.
